# Early one-stage posterior-only surgery for congenital cervicothoracic scoliosis in children: medium- and long-term follow-up

**DOI:** 10.3389/fsurg.2025.1473800

**Published:** 2025-01-23

**Authors:** Cefei Zhang, Fuyun Liu, Ke Xu, Weiming Hu, Bing Xia, Yufeng Zhao

**Affiliations:** Department of Pediatric Orthopedics, The Third Affiliated Hospital of Zhengzhou University, Zhengzhou, China

**Keywords:** congenital scoliosis, cervicothoracic scoliosis, hemivertebra resection, posterior-only, children

## Abstract

**Objective:**

The purpose of this study is to investigate the medium- and long-term correction outcomes and complications of early one-stage posterior-only surgery for congenital cervicothoracic scoliosis in children.

**Methods:**

From March 2006 to March 2022, we retrospectively investigated 33 consecutive cases of congenital cervicothoracic scoliosis treated by one-stage posterior-only surgery, including 15 males and 18 females, with a mean age of 3.2 years. Radiographic parameters, including segmental scoliosis, distal compensatory curve, T1 tilt, clavicle angle, neck tilt, coronal balance distance, segmental kyphosis, and sagittal vertical axis, were measured preoperatively, postoperatively, and at the last follow-up. The results of the measurements were statistically analyzed using paired-sample *t*-tests. Complications were recorded.

**Results:**

The mean operation time was 199.8 min (100–340 min) with an average blood loss of 261.5 ml (80–600 ml). The mean follow-up period was 75.8 months (28–182 months). Fusion levels averaged 3.4 segments (2–6 segments). The segmental scoliosis was improved from 48.2° ± 10.7° preoperatively to 10.0° ± 6.0° postoperatively (*P* < 0.001), with a correction rate of 79.3% ± 11.2%. The distal compensatory curve was spontaneously corrected from 23.4° ± 9.8° preoperatively to 9.2° ± 5.7° postoperatively (*P* < 0.001), with a correction rate of 58.8% ± 19.4%. One case of pleural rupture, three cases of transient nerve root injury, one case of Horner syndrome, and two cases of pleural effusion. Two cases underwent revision surgery due to loss of correction.

**Conclusion:**

Early one-stage posterior-only surgery for congenital cervicothoracic scoliosis in children can effectively correct the local deformities and improve the appearance, and the medium- and long-term correction outcomes are satisfactory. Hemivertebra resection without internal fixation may be considered for some very young children. For the higher level of thoracic hemivertebra, the osteotomy level being shifted down one vertebra is a feasible and safer surgical procedure.

## 1 Introduction

Congenital cervicothoracic scoliosis is a congenital spinal deformity in the lower cervical and upper thoracic spine (C6-T4), with a lower incidence than in the thoracic or lumbar spine (1, 2). The cervicothoracic spine is located between the relatively stationary thoracic vertebrae and the more mobile cervical vertebrae, with relatively concentrated stress, and a slight angle of scoliosis can cause apparent cosmetic abnormalities, such as shoulder imbalance and torticollis. As the deformity progresses, secondary appearance deformities such as head tilt and asymmetric development of the face and eyes can also occur, seriously affecting the patient's appearance and psychological health (3). Conservative treatments, such as brace treatment, have a limited effect on improving the appearance and preventing the progression of deformity. Therefore, early surgical intervention is the only effective treatment strategy (4).

The cervicothoracic junction presents unique anatomical and biomechanical features, making its treatment particularly challenging. Recently, there have been a few reports in the literature regarding treating congenital spinal deformities in the cervicothoracic spine (2, 5–8). However, the studies mentioned above were limited by the older age at surgery and the relatively short follow-up period. The most appropriate time to perform surgery was unclear, and the medium- and long-term correction outcomes in younger children remain uncertain. Therefore, the purpose of the current study is to assess the medium- and long-term correction outcomes and complications of early one-stage posterior-only surgery for congenital cervicothoracic scoliosis in children.

## 2 Materials and methods

Clinical and imaging data of patients with congenital cervicothoracic scoliosis treated by one-stage posterior-only surgery in our hospital between March 2006 and March 2022 were retrospectively reviewed. The following were the inclusion criteria for this research: (1) congenital scoliosis in the cervicothoracic spine (C6-T4), including failure of vertebral formation, failure of vertebral segmentation, or mixed type; (2) treatment with one-stage posterior-only surgery with or without internal fixation; and (3) more than two years of postoperative follow-up. Patients with a spinal correction surgery history or incomplete radiographic data were excluded.

Finally, 33 patients, including 15 males and 18 females, were included in this study, with a mean age of 3.2 years (0.7–13 years). The duration of the operation, intraoperative blood loss, and complications were recorded. This study has been approved by the Ethics Committee of the Third Affiliated Hospital of Zhengzhou University (Ethics Approval Number: 202417001), and informed consent was obtained from all patients' parent or legal guardians.

### 2.1 Surgical technique

All patients underwent one-stage posterior-only surgery, of which 24 patients underwent hemivertebra resection with transpedicular screw fixation, seven patients underwent hemivertebra resection without internal fixation, and two patients with failure of vertebral segmentation underwent convex osteotomy and contralateral bar release combined with pedicle screw internal fixation. Four patients with T1-T2 hemivertebra underwent osteotomy at T2. All surgeries were performed by the same specialist with vast experience in treating congenital spinal deformities.

The patient was placed in the prone position under general anesthesia, and the incision was made layer by layer, peeling off the paravertebral muscles and exposing the subperiosteal vertebral segments above and below the hemivertebra that need to be fused. The hemivertebra's posterior structures were cautiously resected, including the transverse process, lamina, upper and lower facet joints, and the spinous process. The nerve roots above and below the hemivertebra were carefully exposed and protected by rubber strip traction. Intraoperative care was taken to protect the spinal cord, nerve roots, and blood vessels. According to the preoperative design and referring to the 3D-printed spine model, pedicle screws were inserted into the pedicles of the vertebrae above and below the hemivertebra. A temporary rod was placed on the opposite side to stabilize the spine before osteotomy to prevent displacement. The hemivertebra, its cartilage endplates, and the upper and lower discs were exposed, separated, and completely resected until the cancellous bone of the adjacent normal vertebrae, without the remaining hemivertebra and epiphyseal plate. The bar was dissected and released in cases of combined contralateral bar. The ultrasonic scalpel offered unique advantages when working with diastematomyelia. The costovertebral joint and costotransverse joint of the rib attached to the hemivertebra were exposed, and the rib head and the proximal end of the rib were resected to the posterior corner of the rib while avoiding damage to the parietal pleura. The pre-bent connecting rods were placed and rotated to pressurize the convex side and extend the concave side, gradually correcting the scoliosis and kyphosis. When intraoperative fluoroscopy showed the screws and rods were correctly positioned and the spinal deformity was satisfactorily corrected, the resected hemivertebral body and lamina bone were decorticated and placed at the intervertebral space of the fusion segments for autogenous bone grafting. The drainage tube was placed, and the incision was closed layer by layer.

The entire surgical operation was performed under close monitoring of spinal somatosensory evoked potential (SSEP) and motor evoked potential (MEP). A plastic brace or cast vest was recommended to wear for at least 3–6 months after surgery until imaging data showed complete fusion of the bone graft, during which it was adjusted or replaced timely according to the patient's growth rate.

### 2.2 Radiographic assessment

Radiographic parameters, including segmental scoliosis, distal compensatory curve, T1 tilt, clavicle angle, neck tilt, coronal balance distance, segmental kyphosis, and sagittal vertical axis, were measured in posteroanterior and lateral x-ray of the whole spine preoperatively, postoperatively, and at the last follow-up. The method of measurement was described by Chen et al. in 2018 (2). The position and type of the malformation were identified preoperatively by reviewing a cervicothoracic spine computed tomography (CT) scan and three-dimensional reconstruction. A preoperative whole spinal cord magnetic resonance imaging (MRI) was also carried out to determine the intraspinal abnormalities.

### 2.3 Statistical analysis

All parameters were analyzed using SPSS 27.0 statistical software (IBM, USA). Radiographic parameters were presented as mean ± standard deviation. The comparison of radiographic parameters measurements at preoperative, postoperative, and the last follow-up was performed using a paired-sample *t*-tests, and the difference was considered statistically significant at *P* < 0.05.

## 3 Results

The mean operation time was 199.8 min (100–340 min), with an average blood loss of 261.5 ml (80–600 ml). The mean follow-up period was 75.8 months (28–182 months). Fusion levels averaged 3.4 segments (2–6 segments). Fourteen patients had concurrent congenital spinal deformities of other levels, and 16 patients combined with spinal cord malformations (Table 1).

**Table 1:** Demographic and operative data.

Case	Sex	Age (years)	Follow-up (month)	Cervicothoracic deformities^a^	Other spinal deformities^a^	Associated intraspinal anomalies	Fusion levels	Operation time (min)	Blood loss (ml)
1	F	1	145	T1-T2 HV (L), T2 BV, T3 BV, T1-T5 SF (R)	–	Diastematomyelia, tethered cord syndrome, syringomyelia	C7-T5	260	400
2	M	3	108	T1-T2 HV (L), T2-T3 HV (R), T2-T3 SF (L)	–	Diastematomyelia, tethered cord syndrome	T1-T3	310	350
3	M	3	34	C7-T1 HV (L)	–	Spinal meningocele	C7-T1	160	150
4	M	2	28	T1-T2 HV (L)	T4-T5 HV (R), T5-T6 HV (L)	–	T1-T2	140	150
5	F	3	36	T2-T3 HV (L)	T10-T11 HV (R)	–	T2-T3	230	80
6	F	3	128	T1-T2 HV (R)	T7-T8 HV (L)	Spinal meningocele	T1-T2	200	200
7	M	1	118	T1-T2 HV (L)	T10-T11 HV (R), T4-T5 SF (R)	Tethered cord syndrome	T1-T2	180	400
8	F	2	118	T3-T4 HV (R), T3-T5 SF (L)	T5-T6 HV (R)	–	T2-T5	260	420
9	M	2	82	T3-T4 HV (L)	–	–	T3-T4	150	100
10	F	9	39	T2-T3 HV (R), T2-T3 SF (L)	–	Diastematomyelia, tethered cord syndrome	T2-T4	215	300
11	F	0.9	76	T3-T4 HV (L)	–	Diastematomyelia	T3-T5	180	360
12	M	3	157	T3-T4 HV (R)	–	–	T2-T5	165	150
13	F	6	87	T3-T4 HV (L), T3-T5 SF (R)	–	–	T3-T5	240	180
14	F	6	80	T2-T3 HV (L), T2-T5 SF (R)	–	Diastematomyelia, syringomyelia	T1-T5	340	600
15	F	1	65	C6 BV, C7 BV, T3-T4 HV (L), T4-T6 SF (R)	T7 BV	–	T2-T6	230	300
16	M	3	81	T3-T4 HV (L)	T12-L1 HV (R)	–	T3-T4	160	300
17	F	3	154	T4-T6 SF (L)	–	Spinal meningocele, syringomyelia	T4-T7	190	200
18	F	2	182	T1-T4 SF (L)	–	–	T1-T4	165	400
19	F	1	49	T3-T4 HV (R)	L4-L5 HV (L)	–	T3-T4	180	300
20	F	8	28	T2-T3 HV (R), T1-T3 SF (L)	–	–	T1-T4	260	350
21	M	0.7	100	T1-T2 HV (L)	–	Tethered cord syndrome	C7-T3	150	120
22	F	12	51	T3-T4 HV (R), T3-T6 SF (L)	T5 BV	Diastematomyelia	T3-T6	300	310
23	M	13	48	T3-T4 HV (L), C6-T3 SF (R)	–	–	T1-T4	330	350
24	M	1	89	C7 BV, T1-T2 HV (L), T2 BV, T3-T4 HV (L),	T5 BV	–	T2-T4	270	260
25	M	4	29	T1-T2 HV (R), T3-T6 SF (L)	–	Diastematomyelia, syringomyelia	T1-T6	160	260
26	M	2	36	T1-T2 HV (R), T2 BV, T2-T3 HV (R), T1-T4 SF (L)	–	Tethered cord syndrome	T1-T4	300	600
27	F	1	61	T1-T2 HV (L), T2 BV	T5 BV, T9-T10 HV (L)	–	–	100	80
28	M	1	37	T2-T3 HV (L)	–	Spinal meningocele, tethered cord syndrome	–	110	250
29	F	1	49	C7-T1 HV (L), T2-T6 SF (R)	T4-T5 HV (L), T6-T7 HV (L)	Syringomyelia	–	100	130
30	M	2	67	C7 BV, T1-T2 HV (L)	–	–	–	160	150
31	F	2	44	T3-T4 HV (R), T2-T4 SF (L)	–	–	–	130	120
32	F	1	60	C6 BV, T1 BV, C6-T6 SF (R), T3-T4 HV (L)	T4-T5 HV (L)	–	–	140	210
33	M	3	36	T3-T4 HV (L)	T5-T7 SF (R)	Sacral canal cys	–	130	100

^a^ HV, hemivertebra; L, left; R, right; SF, segmentation failure; BV, butterfly vertebra.

**Figure 1:**
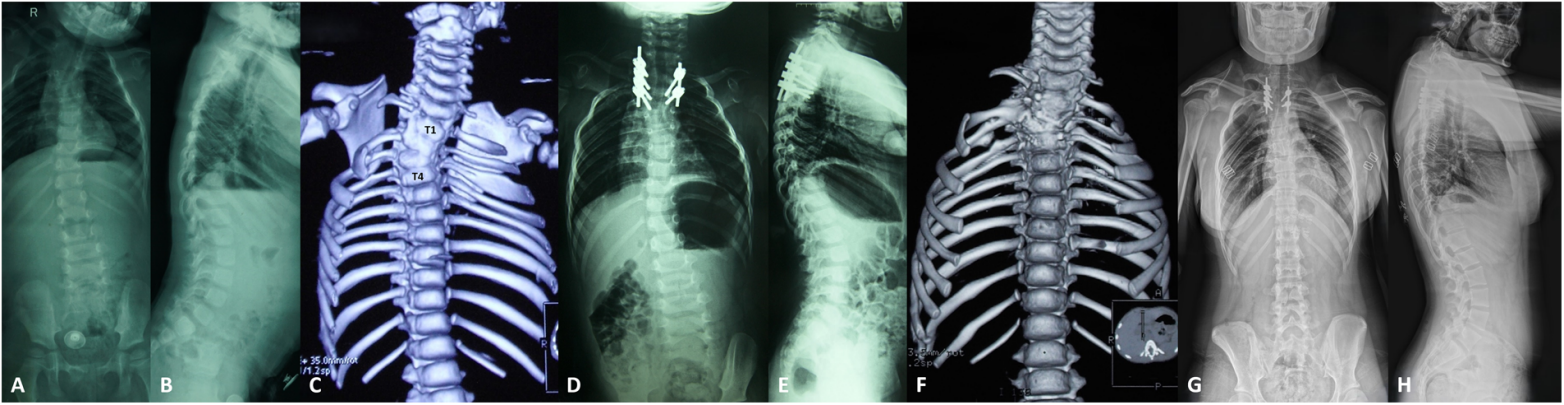
A 2-year-old girl (case 18) with congenital cervicothoracic scoliosis combined with congenital high scapular. **(A,B)** Preoperative radiographs showed that the segmental scoliosis was 54°, the distal compensatory curve was 25°, the T1 tilt was 30°, the clavicle angle was 14°, and the neck tilt was 24°. **(C)** Preoperative CT three-dimensional reconstruction showed the left T1-T4 bar. **(D,E)** Posterior right T2-T3 osteotomy and contralateral bar release combined with pedicle screw internal fixation were performed. Postoperative radiographs showed that the segmental scoliosis was 6° with a postoperative correction rate of 88.9%, the distal compensatory curve was 8°, the T1 tilt was 2°, the clavicle angle was 5°, and the neck tilt was 4°. **(F)** Postoperative CT demonstrated a solid fusion. **(G,H)** At the 15-year follow-up (the patient was 17 years old), the correction was maintained well, and no significant correction loss was observed during the follow-up.

**Figure 2:**
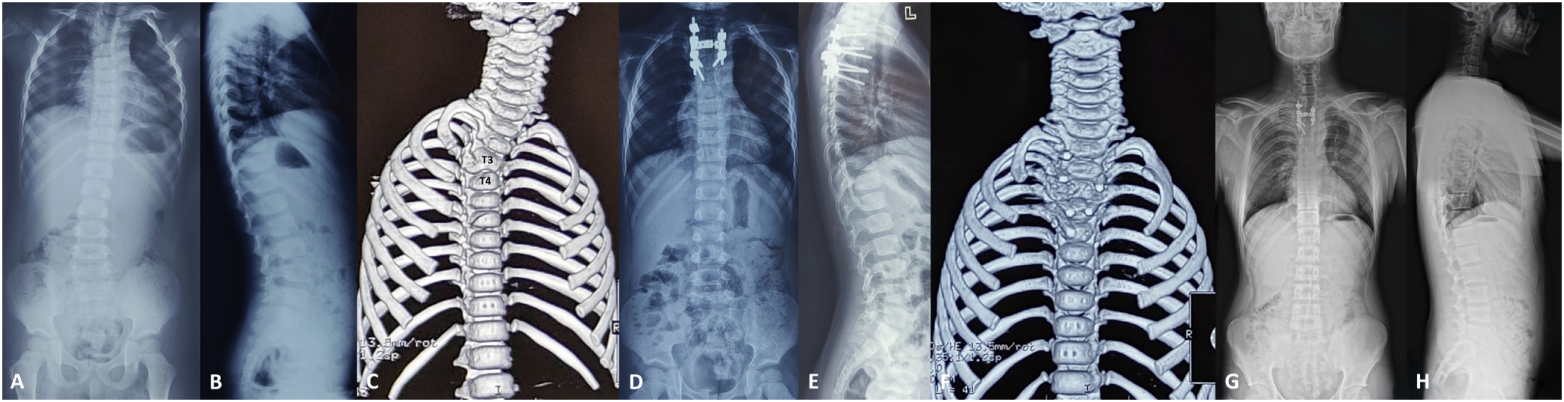
A 3-year-old boy (case 12) with congenital cervicothoracic scoliosis. **(A,B)** Preoperative radiographs showed that the segmental scoliosis was 41°, the distal compensatory curve was 8°, the T1 tilt was 23°, the clavicle angle was 8°, and the neck tilt was 5°. **(C)** Preoperative CT three-dimensional reconstruction showed the right T3-T4 hemivertebra. **(D,E)** Posterior T3-T4 hemivertebra resection combined with pedicle screw internal fixation was performed. Postoperative radiographs showed that the segmental scoliosis was 3° with a postoperative correction rate of 92.7%, the distal compensatory curve was 4°, the T1 tilt was 1°, the clavicle angle was 3°, and the neck tilt was 2°. **(F)** Postoperative CT demonstrated a solid fusion. **(G,H)** At the 13-year follow-up (the patient was 16 years old), the correction was maintained well, and no significant correction loss was observed during the follow-up.

**Figure 3:**
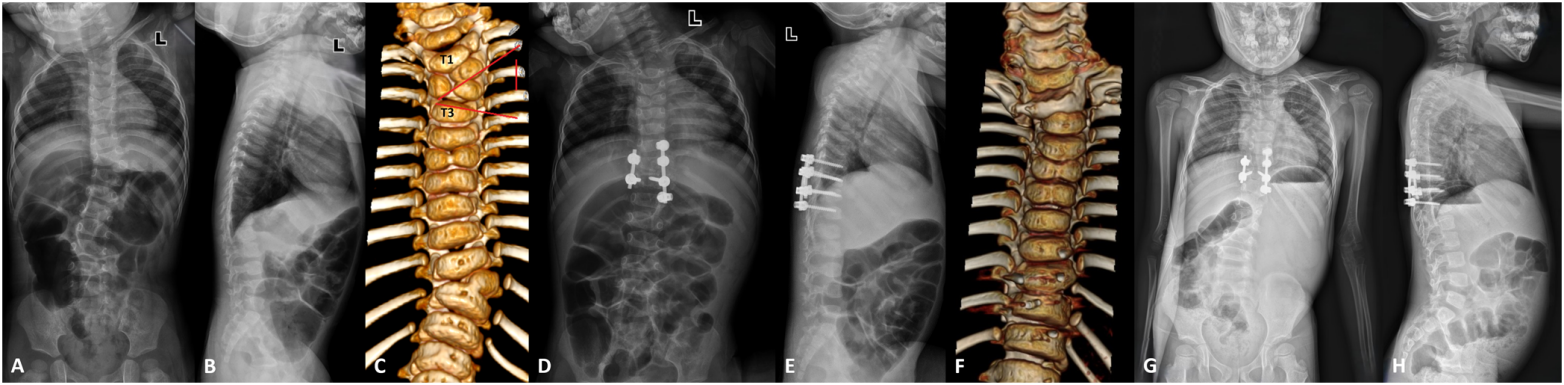
A 1-year-old girl (case 27) with congenital cervicothoracic scoliosis. **(A, B)** Preoperative radiographs showed that the segmental scoliosis was 43°, the distal compensatory curve was 23°, the T1 tilt was 26°, the clavicle angle was 10°, and the neck tilt was 20°. **(C)** Preoperative CT three-dimensional reconstruction showed the left T1-T2 hemivertebra and the T2 butterfly vertebra. **(D,E)** Posterior T2 left hemivertebra resection without internal fixation was performed. Postoperative radiographs showed that the segmental scoliosis was 16° with a postoperative correction rate of 62.8%, the distal compensatory curve was 5°, the T1 tilt was 17°, the clavicle angle was 7°, and the neck tilt was 15°. **(F)** Postoperative CT demonstrated a solid fusion. **(G,H)** At the 5-year follow-up, radiographs showed that the segmental scoliosis was 4° with a correction rate of 90.7%, the distal compensatory curve was 2°, the T1 tilt was 3°, the clavicle angle was 1°, and the neck tilt was 6°. No significant loss of correction was observed during the follow-up.

The radiographic parameters measured preoperatively, postoperatively, and at the last follow-up were summarized in Table 2. The segmental scoliosis was corrected from 48.2° ± 10.7° preoperatively to 10.0° ± 6.0° postoperatively (*P* < 0.001), with a correction rate of 79.3% ± 11.2% (Figures 1–3), and 11.0° ± 7.4° at the last follow-up, which was not statistically different from the postoperative comparison (*P* = 0.355). The distal compensatory curve was spontaneously corrected from 23.4° ± 9.8° preoperatively to 9.2° ± 5.7° postoperatively (*P* < 0.001), with a correction rate of 58.8% ± 19.4%, and 11.4° ± 7.8° at the last follow-up, with no statistically significant difference compared with postoperatively (*P* = 0.080). The segmental kyphosis was corrected from 20.7° ± 8.5° preoperatively to 13.6° ± 6.7° postoperatively (*P* < 0.001) and 11.9° ± 4.4° at the last follow-up, with no significant loss of correction (*P* = 0.114). The T1 tilt, clavicle angle, and neck tilt were significantly improved postoperatively compared with preoperatively (*P* all <0.001), and none of them showed any significant correction loss at the last follow-up (*P* all >0.05).

**Table 2:** Summary of radiographic parameters preoperatively, postoperatively, and at the last follow-up.

	Pre-op	Post-op	Last follow-up	*P*-value (pre-op vs. post-op)	*P*-value (post-op vs. last follow-up)
Coronal plane					
Segmental scoliosis (°)	48.2 ± 10.7	10.0 ± 6.0	11.0 ± 7.4	<0.001	0.355
Distal compensatory curve (°)	23.4 ± 9.8	9.2 ± 5.7	11.4 ± 7.8	<0.001	0.080
T1 tilt (°)	20.9 ± 9.6	7.7 ± 6.0	8.0 ± 7.9	<0.001	0.755
Clavicle angle (°)	7.5 ± 4.0	3.8 ± 2.7	3.1 ± 2.8	<0.001	0.186
Neck tilt (°)	10.9 ± 5.4	4.9 ± 3.6	4.7 ± 3.9	<0.001	0.681
Coronal balance distance (mm)	14.2 ± 9.5	10.0 ± 11.1	13.2 ± 10.2	0.111	0.167
Sagittal plane					
Segmental kyphosis (°)	20.7 ± 8.5	13.6 ± 6.7	11.9 ± 4.4	<0.001	0.114
Sagittal vertical axis (mm)	12.7 ± 30.4	30.8 ± 27.4	0.3 ± 30.3	0.005	<0.001

### 3.1 Complications

In one patient, a pleural rupture occurred during resection of the T3-T4 and T4-T5 hemivertebrae, and a chest drain was left in place intraoperatively and recovered at the time of discharge from the hospital. Three patients suffered from transient nerve root injury, and one patient developed Horner syndrome after surgery. All of them were treated with nutritive neurological drugs, and their symptoms were gradually relieved, and they completely recovered three months after surgery. Two patients had postoperative pleural effusion, which was improved after symptomatic treatment. Two patients underwent revision surgery for residual hemivertebrae and severe progress of the distal compensatory curve, respectively. No pseudoarthrosis, implant loosening, or severe infection occurred during the follow-up period.

## 4 Discussion

Congenital scoliosis (CS) is a spinal deformity due to abnormal vertebral development during the embryonic period, resulting in unbalanced longitudinal growth of the spine (9, 10). CS is primarily progressive and mainly related to the type and location of the deformity, the number of spinal segments involved, and the patient's age (9).

For CS patients with rapid progression and poor prognosis, it is difficult to obtain satisfactory results with conservative treatment, and early surgical treatment is usually advocated to prevent the progression of the local deformities and the development of secondary deformities so that the unaffected portion of the spine can grow normally (10, 11). Otherwise, the spinal deformity will be further aggravated, with increased difficulty in correction, more fusion segments, increased surgical trauma, intraoperative blood loss, and operation time, and a higher risk of neurological injury accompanying it, as well as a more significant financial burden on the patient's family (12). Chang et al. (13) reported that deformity correction with hemivertebra resection with pedicle screw fixation was significantly better in the group of patients before the age of 6 years than the group treated after 6 years of age and had no negative impact on the growth of vertebral body and spinal canal. Nadirov et al. (14) performed a comparative analysis of surgical outcomes for congenital spinal deformities in preschool-age (less than 4 years of age) and primary school-age (6 years of age and older) children. They found that preschool-age children had significantly better corrections than school-age children and that the younger children could achieve full correction with shorter fusion segments. In our study, two patients were operated on at more than 10 years of age (case 22-case 23). The mean operative time and blood loss were 315.0 min and 330.0 ml, respectively, and pleural effusion was found in all patients after the operation.

Due to the high temporal correlation between the development of the spine and the spinal cord, patients with CS often have a combination of spinal cord abnormalities, including diastematomyelia, tethered cord, syringomyelia, and so on (10). Wu et al. (15) reported that 186 (30.3%) of 636 CS patients undergoing surgical treatment were associated with intraspinal anomalies. Wang et al. (5) reported that among 25 patients with congenital cervicothoracic spinal deformities, 6 patients had 8 intraspinal malformations. Such patients should be treated for spinal cord malformations first. Then cervicothoracic spinal deformities or other spinal deformities should be treated simultaneously or in stages according to the patient's conditions under the premise of safety. In this study, 16 patients (48.5%) were found to have combined spinal cord malformations, of which 11 patients underwent surgery for spinal cord malformations at the same time.

Congenital spinal deformities of the cervicothoracic spine tend to be localized short and sharp, more rigid deformities. We believe that preoperative traction is ineffective in such patients, so none of our patients received preoperative traction. Early and timely correction of cervicothoracic spinal deformities can restore balance to the head, neck, and trunk and minimize the impact of spinal deformities on the development of the patient's face and eyes (2). The mean age of our patients was 3.2 years. All of them presented with varying degrees of shoulder imbalance and neck tilt preoperatively, and their appearance was significantly improved after surgery.

Previous literature reported a variety of surgical procedures for congenital cervicothoracic spinal deformities with varying efficacy. Chen et al. (2) treated 18 patients with cervicothoracic hemivertebra using posterior hemivertebra resection, with a postoperative correction rate of 58%. Wang et al. (5) performed 360° osteotomy on 25 patients with congenital cervicothoracic kyphoscoliosis, with a postoperative correction rate of 74.5%. Yu et al. (6) reported a combined anterior and posterior approach for treating congenital cervicothoracic deformities with satisfactory clinical results. Cao et al. (7) described a concave-side distraction technique for correcting congenital cervicothoracic scoliosis, with a postoperative correction rate of 66.7%. Zhang et al. (8) proposed a posterior correction and fusion alone without hemivertebra osteotomy for congenital cervicothoracic spinal deformities, with a postoperative correction rate of 76%. All patients in this study underwent one-stage posterior-only surgery, of which 24 patients underwent hemivertebra resection with transpedicular screw fixation, seven patients underwent hemivertebra resection without internal fixation, and two patients with failure of vertebral segmentation underwent convex osteotomy and contralateral bar release combined with pedicle screw internal fixation. They all achieved satisfactory therapeutic results after surgery, with a postoperative correction rate of 79.3%.

Posterior hemivertebra resection with transpedicular screw fixation is recognized as a safe and effective procedure for CS caused by hemivertebra, which can remove the causative factors directly (10, 11, 16). However, pedicle fracture and implant loosening may occur in children because the skeleton is not fully developed. For tiny toddlers, choosing the appropriate internal fixation screws might be challenging. In addition, the cervicothoracic junction is a stress-concentrated region, and the narrow pedicles of the cervicothoracic spine in children are often combined with various abnormal vertebral structures, making it extremely difficult to place the pedicle screw (8). Hemivertebra resection without internal fixation can avoid implant-related complications. Xia et al. (17) performed posterior hemivertebra resection without internal fixation in 16 young CS patients. After an average follow-up of 7 years, the correction effect was satisfactory, with no significant loss of correction. In this study, seven patients underwent hemivertebra resection without internal fixation (case 27-case 33), with a mean age of 1.6 years, mean operation time, and intraoperative blood loss of 124.3 min and 148.6 ml, respectively, and satisfactory correction was achieved after surgery.

The cervicothoracic junction has complex anatomical structures adjacent to essential nerves, blood vessels, and organs and is often combined with neurovascular malformations, which can easily cause neurovascular injuries during surgery in this region (2, 8). Forming the brachial plexus involves the C7-T1 nerve roots, and sensory-motor deficits in the upper extremities can occur if damaged (8). In addition, there is a particular complication, Horner syndrome, which occurs in association with damage to the oculo-sympathetic nerve pathway, the fibers of which originate in the posterolateral hypothalamus and descend uncrossed in the cervical region to the lateral midbrain, pons, medulla, and spinal cord, reaching the C8-T2 spinal cord center (18). Chen et al. (2) reported that 18 patients with cervicothoracic hemivertebra developed Horner syndrome in one patient and transient right arm radicular pain in one patient after surgery. Wang et al. (5) reported Horner syndrome in one patient and transient nerve root injury in 11 patients after surgery in 25 patients with congenital cervicothoracic kyphoscoliosis. In our series, one patient developed Horner syndrome postoperatively, and three patients developed transient nerve root injury postoperatively. The osteotomy process causes most intraoperative nerve injuries. Therefore, we propose that the osteotomy level be shifted down one vertebra, thus avoiding neurovascular injury and making the procedure safer. In this study, four patients with T1-T2 hemivertebra underwent osteotomy at T2 (case 24-case 27), with satisfactory postoperative correction, no neurovascular injury, and no appreciable corrective loss throughout the follow-up.

There were several limitations to this study. First, the number of patients in this study was relatively small, so further studies with larger samples are needed. Second, most younger patients still have spinal growth potential, and longer-term follow-up observations are required. Third, this study did not include results on the quality of life and satisfaction of patients and parents during follow-up, and further studies are needed in the future. Additionally, there may be potential bias due to the lack of stratification on the subtypes of CS due to the limited size of the population.

## 5 Conclusion

Early one-stage posterior-only surgery for congenital cervicothoracic scoliosis in children can effectively correct the local deformities and improve the appearance, with less trauma, fewer fusion segments, maximum preservation of spinal growth potential, and the medium- and long-term correction outcomes are satisfactory. Hemivertebra resection without internal fixation may be considered for some very young children. For the higher level of thoracic hemivertebra, the osteotomy level being shifted down one vertebra is a feasible and safer surgical procedure, thus avoiding neurovascular damage.

## Data Availability

The original contributions presented in the study are included in the article/Supplementary Material, further inquiries can be directed to the corresponding author.
